# Rheumatism as a Cause of Epistaxis in Children

**Published:** 1904-07-23

**Authors:** 


### RHEUMATISM AS A CAUSE OF EPISTAXIS IN CHILDREN.

THE study of rheumatic manifestations, as these occur in the early years of life, has effected something like a revolution in the clinical presentation of the disease as compared with the teaching generally prevalent, say, a generation ago. Instead of being regarded as mainly an affection of the joints, with a greater or less liability to cardiac valve disease—the picture presented by the rheumatic adult —acute rheumatism is seen, when studied in the child, to have a much wider and more varied range of phenomena. Erythemas, tonsillitis, chorea, purpura, subcutaneous nodules, perhaps also pleurisy and other localised inflammatory processes, are now commonly recognised as members of the rheumatic series, and are more frequent as exhibitions of the disease in early life than the acute polyarthritis which is the limited display of the disease to which the adult is mainly liable. The tendency is to add to the number of incidents having a rheumatic basis. Recently[1] attention was directed in these columns to abdominal pain in children as an ex-expression of rheumatism. It is also to be noted that the same claim has been advanced for epistaxis. Dr. Sydney Phillips[2] was one of the earliest observers to draw attention to this point. He published a series of cases, in some of which the epistaxis came on with an attack of acute rheumatism, whilst in others the bleeding occurred in the intervals between articular inflammations, chorea, or other recognised rheumatic incidents. Dr. Phillips suggested that in every case of epistaxis in early life care should be taken to examine the joints, as undoubtedly in children synovial effusion and articular rheumatism may occur without pain. It has been charged against the salicylates that they sometimes cause epistaxis; but, in view of the above records and of the fact that none of the patients had taken salicylates prior to the epistaxis, it becomes a question whether the result attributed to the remedy ought not to be debited to the disease. Dr. Graham Langwill[3] has also described attacks of epistaxis in rheumatic children. In one of his cases the bleeding occurred at each of three separate attacks of acute rheumatism. There cannot be much difficulty in accepting the clinical position suggested by these records. If rheumatism, as few deny, is capable of producing subcutaneous hæmorrhages as in *purpura rheumatica* it is not difficult to conceive that similarly it may produce epistaxis. And it may be that the epistaxis which is occasionally noted in cases of mitral obstruction, and is then attributed to the valvular lesion, has a more direct relationship to the rheumatic process. In a recent article Dr. Herbert Tilley[4] accepts the position that epistaxis may be in children an expression of rheumatism, and claims that treatment by salicylates is successful in preventing recurrence.

[1] The Hospital, June 18, 1904. [2] The Lancet, Feb. 22, 1902. [3] Scottish Med. & Surg. Jour., Nov. 1903. [4] Clin. Jour., June 1, 1904.

